# Association between Salt Intake and Albuminuria in Normotensive and Hypertensive Individuals

**DOI:** 10.1155/2013/523682

**Published:** 2013-09-19

**Authors:** Arsalan Khaledifar, Mojagn Gharipour, Ahmad Bahonar, Nizal Sarrafzadegan, Alireza Khosravi

**Affiliations:** ^1^Cardiology Department, School of medicine, Shahrekord University of Medical Sciences, Shahrekord, Iran; ^2^Isfahan Cardiovascular Research Center, Isfahan Cardiovascular Research Institute, Isfahan University of Medical Sciences, Isfahan, Iran; ^3^Hypertension Research Center, Isfahan University of Medical Sciences, Isfahan, Iran; ^4^Interventional Cardiology Research Center, Isfahan Cardiovascular Research Institute, Isfahan University of Medical Sciences, Isfahan, Iran; ^5^Hypertension Research Center, Isfahan Cardiovascular Research Institute, Isfahan University of Medical Sciences, Isfahan, Iran

## Abstract

*Background*. There is a little published data regarding the association between salt intake and albuminuria as an important alarm for progression of cardiovascular and renal dysfunction. We aimed to assess this relationship to emphasize the major role of restricting salt intake to minimize albuminuria and prevent these life-threatening events. *Methods*. The study population comprised 820 individuals. Participants were assigned to groups as follows: normal albuminuria, slight albuminuria, and clinical albuminuria. Daily salt intake was assessed on the basis of 24-hour urinary sodium excretion, since urinary sodium excretion largely equals sodium intake. *Results*. In normotensive participants, the mean level of urine albumin was higher in those who had higher amounts of salt intake with a significantly upward trend (the mean urinary albumin level in low-salt-diet group, in medium-salt-intake group, and in high-salt-intake group was 42.70 ± 36.42, 46.89 ± 38.91, and 53.38 ± 48.23, resp., (*P* = 0.017)). There was a significant positive correlation between 24-hour urinary sodium secretion and the level of urine albumin (beta = 0.130, *P* < 0.001). The amount of salt intake was significantly associated with urine albumin concentration (beta = 3.969, SE = 1.671, *P* = 0.018). *Conclusion*. High salt intake was shown to be associated with higher level of microalbuminuria even adjusted for potential underlying risk factors.

## 1. Introduction

Control of dietary intake and its main components is a main and essential strategy to prevent progression of cardiovascular disorders as left ventricular hypertrophy and kidney diseases as renal fibrosis. In this regard, slowing progression of renal diseases forward to end stages is obtained by proper control of blood pressure and its regulative mechanisms. In this regard and because of the partially low efficacy of medications to present kidney disease progression, dietary behavioral modification can play a major role in this goal [1, 2]. For decades, association between salt intake and blood pressure has been discussed, and it has been shown that the increase of salt intake contributes to the prevalence of hypertension. However, relationship between high salt intake and albuminuria has been already questioned [3, 4].

Although albuminuria is not a usual finding in general population, according to the Third National Health and Examination Survey (NHANES III) [5], about 7.8% of individuals suffered from this abnormal phenomenon so that it frequently appeared in higher risk groups including diabetics and hypertensive patients. In addition, those with microalbuminuria are at the greatest risk for progression to both cardiovascular and renal events [6]. Recently, it has been shown that the reducing proteinuria is associated with a reduction in both renal and cardiovascular events [7]. Besides, it is now clear that reducing dietary salt consumption can be consider as a potentially modifiable risk that may protect patients from cardiovascular and renal disease progression [8]. However, to date, there have been a few syntheses of the existing evidence regarding the association between salt intake and albuminuria as an important alarm for progression of cardiovascular and renal dysfunction. Therefore, we aimed to assess this relationship to emphasize the major role of restricting salt intake to minimize albuminuria and prevent these life-threatening events.

## 2. Methods

The study population comprised 820 normotensive individuals (457 men and 363 women), ages 19–81 years (median 35 years) who participated in an Isfahan, Iran population-based survey [2]. All participants gave informed consent, and procedures followed were in accordance with the Ethical Committee of Isfahan University of Medical Sciences. Baseline information regarding demographics, educational level, medical history, and medications was recorded. Height, weight and waist circumference were measured on the day of the visit to the outpatient clinic. Body mass index (BMI) was calculated as weight was divided by height squared (kg/m^2^). Blood pressure was measured twice in the left arm by an examining physician using a mercury column sphygmomanometer (Korotkoff phases I and V) after the subject had been at rest in the seated position for 5 minutes. Hypertension was defined as a systolicblood pressure (SBP) of ≥140 mmHg, a diastolic blood pressure (DBP) of ≥90 mmHg, or those who were receiving antihypertensive therapy at the time of the examination [4]. Smoking status was also considered as smoking ≥1 cigarette per day in the year preceding the examination. Participants fasted from the evening before the interview, and on the day of interview a first voided urine sample was collected into a sterile container for albumin estimation. Blood was also drawn after an 8–12 overnight fasting period in the morning after completion of the 24-hour urine collection. Plasma biochemical indices including sodium and potassium concentrations, as well as BUN and serum creatinine levels were measured by standard laboratory procedures. Urinary albumin concentrations were determined by immunoturbidixnetric assay, and the participants were assigned to groups as follows: normal albuminuria (men ≤ 28 mg/L, women ≤ 29 mg/L); slight albuminuria (men 29–299 mg/L, women 30–299 mg/L); and clinical albuminuria (≥300 mg/L) [9]. Daily salt intake was assessed on the basis of 24-hour urinary sodium excretion (UNaV), since urinary sodium excretion largely equals sodium intake, when people are in steady state [10]. The subjects were divided into tertiles according to the level of 24-hour urinary sodium excretion: low-salt-intake group (*n* = 273, urine sodium ≤ 132 mmol/24 h), medium-salt-intake group (*n* = 271, urine sodium: 133–186 mmol/24 h), and high-salt-intake group (*n* = 276, urine sodium ≥ 187 Bmmol/24). 

Results were presented as mean ± standard deviation (SD) for quantitative variables and summarized by absolute frequencies and percentages for categorical variables. Continuous variables were compared using *t* test or one-way analysis of variance (ANOVA) and/or nonparametric Mann-Whitney *U* test or Kruskal-Wallis test whenever the data did not appear to have normal distribution or when the assumption of equal variances was violated across the three groups of TR. Categorical variables were, on the other hand, compared using chi-square test or Fisher's exact test when more than 20% of cells with expected count of less than 5 were observed. Correlation between quantitative variables was assessed using Pearson's correlation coefficient test. For the statistical analysis, the statistical software SPSS version 19.0 for windows (SPSS Inc., Chicago, IL) and the statistical package SAS version 9.1 for windows (SAS Institute Inc., Cary, NC, USA) were used. *P* values of 0.05 or less were considered statistically significant.

## 3. Results

Comparing baseline characteristics and clinical data across the three groups of salt intake (Table 1) revealed that male gender distribution was more in low-salt-intake group. The participants in lower-salt intake categories were significantly older, and had lower BMI and waist circumference. No discrepancy was observed in overall prevalence of hypertension, current smoking, and also in mean systolic and diastolic blood pressures. Regarding laboratory indices, the average urine creatinine level and urine albumin concentration were both higher in those who had higher salt intake.

**Figure 1:**
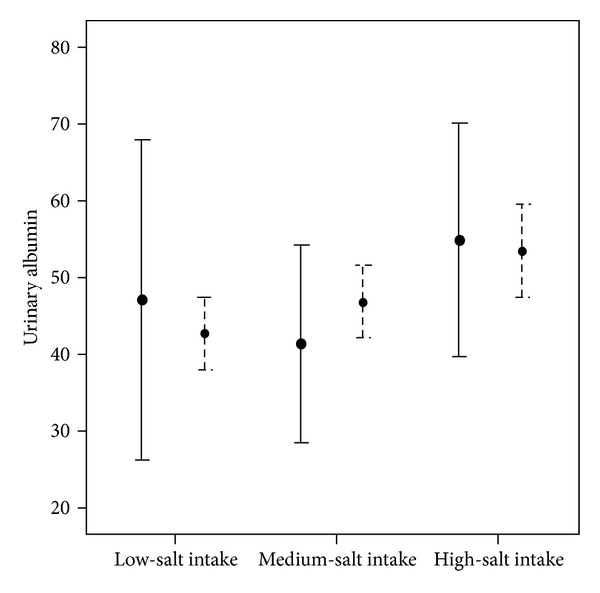
Mean urine albumin level in different salt intake groups.

According to the classification of albuminuria, 42.4% of individuals had normal range of urine albumin level, 57.4% had slight albuminuria, and only 0.1% suffered from clinical albuminuria. As presented in Figure 1, in normotensive participants, the mean level of urine albumin was higher in those who had higher amounts of salt intake with a significant upward (that the mean urinary albumin level in low-salt-diet group was 42.70 ± 36.42; in medium-salt-intake group, 46.89 ± 38.91; and in high-salt-intake group, 53.38 ± 48.23 (*P* = 0.017)), while in this trend the changes were not significant in hypertensive ones (mean urinary albumin level in low-salt-diet group was 47.09 ± 38.25, in medium-salt-intake group was 41.35 ± 24.96, and in high-salt-intake group was 54.85 ± 43.50, *P* = 0.529).

**Figure 2:**
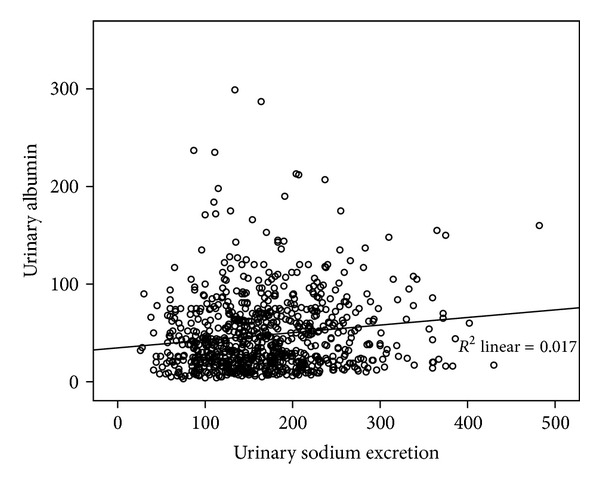
Correlation between 24-hour urinary sodium secretion and level of urine albumin (beta = 0.130, *P* < 0.001).

There was a significant positive correlation between 24-hour urinary sodium secretion and the level of urine albumin (beta = 0.130, *P* < 0.001) (Figure 2). Using a multivariable linear regression model (Table 2) and with the presence of baseline variables, the amount of salt intake was significantly associated with urine albumin concentration (beta = 3.969, SE = 1.671, *P* = 0.018).

## 4. Discussion

Positive or inverse association between salt intake and albuminuria has been also unknown. Although some evidence showed that low daily salt intake is associated with albuminuria in diabetic patients [11], some others showed that high salt intake increases blood pressure and albuminuria in diabetic patients that is associated with insulin resistance and increased glomerular pressure [12, 13]. In the studies on animal models, high sodium treatment led to a significantly increased excretion of albumin in the urine of animals compared with control animals and also animals on normal drinking water [14]. It has been also demonstrated, in line with increased albuminuria following high salt intake, that the activation of inflammatory processes can occur by increase of salt intake and that both the increase of the urinary level of albumin and increased inflammation can trigger end-stage renal disease [15]. Microalbuminuria is an important alarm indicating a defected blood urine interface that may represent a serious diffuse vascular disease throughout the circulation [7, 8] Hence, presence of microalbuminuria can help the clinician to identify those individuals with greater cardiovascular and renal risk factors and a greater need for improved other related risk profile including blood pressure, lipids, insulin resistance, and hyperglycemia.

Our findings can be assessed through different aspects. First, we showed a direct association between high salt intake and the increase of albuminuria that is consistent with some previous observations. Also, this association was also shown independent from other underlying risk factors such as hypertension and smoking. Moreover, we considered only nondiabetic patients to inhibit interactive effects of diabetes and insulin resistance as a trigger for albuminuria. 

Our results are based on this mechanism that high sodium is harmful to the selective permeability of the glomerular basement membrane and worsens the urinary excretion of albumin [16]. It is now believed that the glomerular podocyte in the outer layer of glomerular epithelial cells is the cell primarily responsible for the prevention of albuminuria in health state, and thus podocyte damages underlie albuminuria [17]. This tissue damage can be mediated by the secretion of some inflammatory mediators such as transforming growth factor-*β* (TGF-*β*) and cytokines produced in response to changes in systemic factors, particularly blood pressure [18]. Besides, the increase in blood pressure during high sodium intake can raise the activity of TGF-*β* and therefore increase the urinary albumin excretion [18]. This mechanism can successfully explain our finding. However, an unusual finding in the present study was the association between salt intake and albuminuria only in normotensive individuals, not in hypertensive ones that should be tested in further studies with considering different hypertension categories, using antihypertensive drugs and other confounding factors related to this index.

In conclusion, high salt intake was shown to be associated with higher level of microalbuminuria even adjusted for potential underlying risk factors. Although this association seems to be related to hypertension state, it may be more evidenced in normal blood pressure condition.

## Figures and Tables

**Table 1:** Baseline characteristics and clinical data of study population.

Characteristics	low-salt-intake (*n* = 273)	med-salt-intake (*n* = 271)	high-salt-intake (*n* = 276)	*P* value
Male gender	190 (69.6)	137 (50.6)	130 (47.1)	<0.001
Age, yr	38.78 ± 14.00	35.88 ± 11.51	37.07 ± 11.97	0.029
Body mass intake (kg/m^2^)	24.93 ± 4.22	25.10 ± 4.56	26.43 ± 4.25	<0.001
Waist circumference (cm)	80.60 ± 11.44	82.04 ± 11.93	85.15 ± 12.45	<0.001
Current smoker	25 (9.4)	29 (11.3)	22 (8.5)	0.156
History of hypertension	23 (8.4)	17 (6.3)	34 (12.3)	0.060
Systolic PB, mmHg	106.71 ± 12.82	105.83 ± 12.56	107.45 ± 13.67	0.351
Diastolic BP, mmHg	71.23 ± 9.36	70.54 ± 9.55	72.34 ± 10.68	0.101
Serum BUN level	15.31 ± 3.37	15.21 ± 3.55	15.12 ± 3.73	0.812
Serum creatinine level	0.94 ± 0.17	0.98 ± 0.23	0.98 ± 0.21	0.052
Serum sodium level	138.96 ± 3.12	139.11 ± 3.22	139.25 ± 3.23	0.551
Urine creatinine level	119.24 ± 46.61	133.82 ± 44.21	145.66 ± 49.54	<0.001
Urine albumin	43.07 ± 37.48	46.55 ± 38.18	53.56 ± 47.60	0.011

**Table 2:** Association between salt intake and albuminuria in a linear regression model.

Variable	*P* value	Beta	SE
Salt intake	0.018	3.969	1.671
Gender	0.649	1.384	3.040
Age	0.520	0.074	0.115
Body mass index	0.240	0.399	0.339
Hypertension	0.423	4.348	5.428
Smoking	0.002	−7.233	2.355
Systolic BP	0.139	0.291	0.196
Diastolic BP	0.321	−0.236	0.238
